# Moxifloxacin HCl-Incorporated Aqueous-Induced Nitrocellulose-Based In Situ Gel for Periodontal Pocket Delivery

**DOI:** 10.3390/gels9070572

**Published:** 2023-07-13

**Authors:** Setthapong Senarat, Catleya Rojviriya, Katekeaw Sarunyakasitrin, Juree Charoentreeraboon, Wiwat Pichayakorn, Thawatchai Phaechamud

**Affiliations:** 1 Programme of Pharmaceutical Engineering, Faculty of Pharmacy, Silpakorn University, Nakhon Pathom 73000, Thailand; senarat_s@silpakorn.edu; 2 Synchrotron Light Research Institute, Mueang District, Nakhon Ratchasima 30000, Thailand; catleya@slri.or.th; 3 Secretary Office of Faculty, Faculty of Pharmacy, Silpakorn University, Nakhon Pathom 73000, Thailand; sarunyakasitrin_k@su.ac.th; 4 Natural Bioactive and Material for Health Promotion and Drug Delivery System Group (NBM), Faculty of Pharmacy, Silpakorn University, Nakhon Pathom 73000, Thailand; 5 Pharmaceutical Intellectual Center “Prachote Plengwittaya”, Faculty of Pharmacy, Silpakorn University, Nakhon Pathom 73000, Thailand; charoenteeraboo_j@su.ac.th; 6 Department of Industrial Pharmacy, Faculty of Pharmacy, Silpakorn University, Nakhon Pathom 73000, Thailand; phaechamud_t@su.ac.th; 7 Department of Pharmaceutical Technology, Faculty of Pharmaceutical Sciences, Prince of Songkla University, Songkhla 90110, Thailand; wiwat.p@psu.ac.th

**Keywords:** moxifloxacin HCl, nitrocellulose, in situ gel, periodontal pocket, delivery

## Abstract

A drug delivery system based on an aqueous-induced in situ forming gel (ISG) consists of solubilizing the drug within an organic solution of a polymer using a biocompatible organic solvent. Upon contact with an aqueous medium, the solvent diffuses out and the polymer, designed to be insoluble in water, solidifies and transforms into gel. Nitrocellulose (Nc), an aqueous insoluble nitrated ester of cellulose, should be a promising polymer for an ISG using water induction of its solution to gel state via phase inversion. The aim of this investigation was to develop and evaluate a moxifloxacin HCl (Mx)-incorporated aqueous-induced Nc-based ISG for periodontitis treatment. The effects of different solvents (*N*-methyl pyrrolidone (NMP), DMSO, 2-pyrrolidone (Py), and glycerol formal (Gf)) on the physicochemical and bioactivity properties of the ISGs were investigated. The viscosity and injection force of the ISGs varied depending on the solvent used, with Gf resulting in higher values of 4631.41 ± 52.81 cPs and 4.34 ± 0.42 N, respectively. All ISGs exhibited Newtonian flow and transformed into a gel state upon exposure to the aqueous phase. The Nc formulations in DMSO showed lower water tolerance (12.50 ± 0.72%). The developed ISGs were easily injectable and demonstrated water sensitivity of less than 15.44 ± 0.89%, forming a gel upon contact with aqueous phase. The transformed Nc gel effectively prolonged Mx release over two weeks via Fickian diffusion, with reduced initial burst release. Different solvent types influenced the sponge-like 3D structure of the dried Nc ISGs and affected mass loss during drug release. Incorporating Nc reduced both solvent and drug diffusion, resulting in a significantly narrower zone of bacterial growth inhibition (*p* < 0.05). The Mx-incorporated Nc-based ISGs exhibited efficient antibacterial activity against four strains of *Staphylococcus aureu* and against periodontitis pathogens including *Aggregatibacter actinomycetemcomitans* and *Porphyromonas gingivalis*. This study suggests that the developed Mx-incorporated Nc-based ISGs using DMSO and NMP as the solvents are the most promising formulations. They exhibited a low viscosity, ease of injection, and rapid transformation into a gel upon aqueous induction, and they enabled localized and prolonged drug release with effective antibacterial properties. Additionally, this study represents the first reported instance of utilizing Nc as the polymer for ISG. Further clinical experiments are necessary to evaluate the safety of this ISG formulation.

## 1. Introduction 

**Figure 1:**
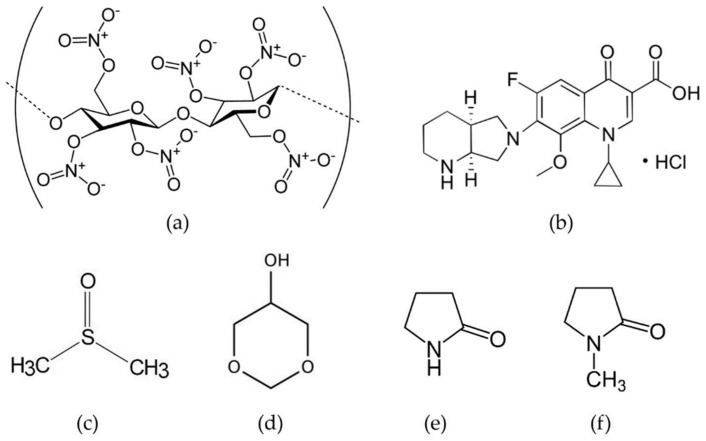
Chemical structures of nitrocellulose (Nc) (**a**), moxifloxacin HCL (Mx) (**b**), dimethyl sulfoxide (DMSO) (**c**), glycerol formal (Gf) (**d**), 2-pyrrolidinone (Py) (**e**), and *N*-methyl-2-pyrrolidone (N) (**f**).

Nitrocellulose (Nc), a nitrated ester of cellulose, is a white, fibrous, pulp-like substance which has no odor or taste and has the formula C_12_H_16_N_4_O_18_ (Figure 1a). Due to its explosive nature when dry, NC is typically kept wet using water or organic solvents like isopropyl alcohol, esters, ketones, or glycol ethers [1]. NC can be classified based on various properties, primarily its nitrogen content, which affects its fundamental characteristics [2]. As the nitrogen content increases, the density of NC also increases [3]. Moreover, there is a corresponding increase in intrinsic viscosity with higher levels of nitration [4]. In chronic, sub-chronic, and multigeneration studies conducted on rats and dogs, NC was found to be non-toxic. However, in mice, mortality was observed, which was attributed to the size of the fibers in relation to the murine intestinal lumen rather than chemical toxicity [1,5]. NC has proven to be versatile and beneficial in numerous fields. It is used as a protective covering to prevent wound contamination, in cosmetic preparations, for film production, as an inert support in chromatographic separations, and in the manufacturing of lacquers, artificial leathers, adhesives, and other products [1,5,6,7]. It has also been employed as a membrane filter impregnated with silver nanoparticles for purifying drinking water [8]. In medical applications, the exceptional biological and physiochemical properties of NC have led to its utilization in the form of a paper-like matrix with microscale porous pores known as NC membrane. The NC membrane is frequently used to immobilize nucleic acids in Southern and Northern blots, as well as proteins in Western blots [7,9,10]. Furthermore, NC bandages have proven effective in wound healing, particularly for hard-to-cover wounds. Nano-porous NC liquid bandages have been developed, exhibiting enhanced antibacterial effects, accelerated healing time, and non-toxicity to the wound itself [11]. NC has demonstrated itself as a critical substance with unique features that holds potential for various sectors. However, the application of an NC-based in situ forming depot as a drug delivery system for periodontitis treatment has not been previously explored, making it an interesting avenue for development.

The aqueous-induced phase-inversion-based in situ forming gel (ISG) system is currently recommended for the delivery of antimicrobial drugs in periodontal pockets [12,13]. Initially, it comprises a polymeric solution containing the drug, which transforms into a gel-like or solid matrix after injection and exposure to the aqueous fluid in the periodontal pocket during periodontitis treatment [14,15,16]. Atridox^®^ and Atrisorb-D^®^ FreeFlow^TM^ (TOLMAR Inc. Fort Collins, CO, USA) are licensed ISG products loaded with doxycycline hyclate, providing local inhibition of bacterial growth and facilitating healing in periodontitis through controlled drug release [17].

Moxifloxacin HCl (Mx)(C_21_H_24_FN_3_O_4_•HCl) (Figure 1b) is a fourth-generation fluoroquinolone antibiotic with broad-spectrum activity against Gram-positive, Gram-negative, and anaerobic bacteria [18,19]. It acts by inhibiting the bacterial enzymes DNA gyrase (topoisomerase II) and topoisomerase IV, resulting in the inhibition of DNA replication and cell death in susceptible bacterial species [20]. In ocular applications, Mx-loaded poly(lactic-co-glycolic acid) (PLGA) microparticles have been encapsulated in a chondroitin sulfate-based bio-adhesive to localize drug release through in situ gelation, potentially combining antibiotic prophylaxis and wound healing in the eye [21]. Mx-loaded bilosomes incorporated into a gel-based system containing sodium deoxycholate, Cremophor EL, and Span 60 have shown increased corneal residence time, improved drug permeation, and efficient antimicrobial activity [22]. In situ gelling microemulsions have also been used to enhance Mx permeation in intraocular surgery [23].

Thermosensitive in situ gel formulations incorporating Mx have been developed in which the Mx retained its antimicrobial activity in this gel for periodontitis treatment [20]. Furthermore, Mx has been loaded into in situ gel-forming eye drops using sodium alginate as a gelling agent in combination with hydroxypropyl methylcellulose (HPMC) as a viscosity-enhancing agent for ocular infection treatment [24]. Antimicrobial studies have demonstrated that Mx maintains its activity when formulated as an in situ forming gel for periodontal drug delivery, effectively targeting selected strains of *S. aureus* and *E. coli*. These findings indicate the potential of Mx as an active compound in periodontal drug delivery systems [21,24]. Additionally, Mx-loaded dental implants have been fabricated using the solvent casting technique with ethyl cellulose and other copolymers. These formulations have exhibited significant in vitro antibacterial activity against *Streptococcus mutans* for up to 6 days, retaining their antibacterial efficacy [25]. However, the use of Mx-loaded solvent-removal phase-inversion-induced in situ forming gel (ISG) for periodontitis treatment, utilizing NC as the polymeric matrix, has not been previously reported.

Currently, solvent-removal phase-inversion-induced in situ forming gel (ISG) systems commonly utilize dimethyl sulfoxide (DMSO) (Figure 1c), glycerol formal (Gf) (Figure 1d), 2-pyrrolidinone (Py) (Figure 1e), and *N*-methyl-2-pyrrolidone (N) (Figure 1f) as solvents. These solvents are preferred due to their established use in the pharmaceutical industry [26]. NMP is widely present in various commercial pharmaceutical products, and the European Commission Scientific Committee on Consumer Safety has determined that it has low acute toxicity when administered orally, dermally, or by inhalation [27,28,29]. DMSO is approved by the US FDA for the treatment of interstitial cystitis [30,31]. Studies have shown that DMSO is highly soluble in water and can disrupt the water structure, forming strong hydrogen bonds with water molecules due to its stronger interaction with water [30,31]. Vancomycin HCl-loaded fatty acid in situ forming matrices have been successfully developed using DMSO as a solvent for antimicrobial inhibition in patients with joint infection after total knee arthroplasty [32] and periodontitis [33]. Py is used as a solvent in in situ forming gels due to its biocompatibility and low toxicity (LD_50_ Rat oral 6.5 g/kg) [34,35]. It has also been reported as a plasticizer to improve the mechanical properties and reduce drug burst release in spider silk films [36]. Studies have shown that when DMSO, NMP, or Py were used in in situ forming implants made with PLA/PLGA or PLA, the initial drug release decreased in the order of DMSO > NMP > Py [37]. Although there are limited studies on the use of glycerol formal in ISM systems, it has been reported as a solvent for doxorubicin-loaded zein in situ gels for interstitial chemotherapy of colorectal cancer [38] and pingyangmycin hydrochloride-loaded zein/zein–sucrose acetate isobutyrate (SAIB) in situ gels for treating venous malformations [39]. In this study, these four solvents, DMSO, Gf, Py, and N, were evaluated as solvents for the development of Mx-loaded solvent-removal phase-inversion-induced NC-based ISG systems.

Developing a new drug delivery system for localized application in the deep path of the periodontal pocket region poses challenges due to the constant flow of saliva and gingival crevicular fluid. Gels have emerged as an interesting localized vehicle for the effective delivery of therapeutic agents [40]. In this study, the preparation of Mx-loaded solvent-removal phase-inversion-induced NC-based ISG systems was attempted for delivery to the periodontal pocket. The fluid properties, injectability, matrix formation behavior, drug release, and release kinetics of the systems were evaluated. Additionally, the topography of the dried NC matrix was examined using scanning electron microscopy and X-ray tomography. The antimicrobial activities of the systems against various strains of *S. aureus* and periodontal pathogens, including *Porphyromonas gingivalis* and *Aggregatibacter actinomycetemcomitans*, were investigated.

## 2. Results and Discussion

**Figure 2:**
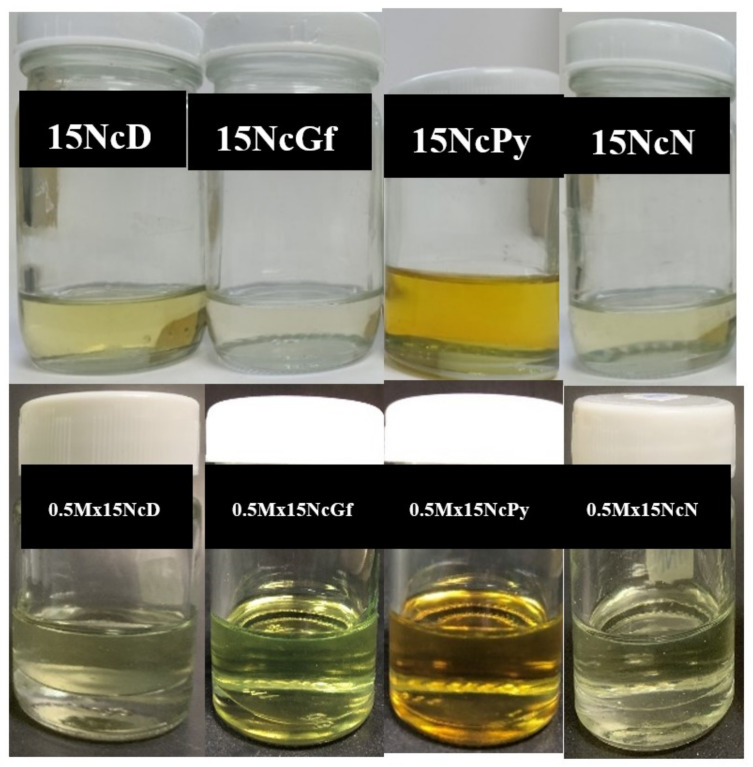
Physical appearance of 15% *w*/*w* Nc solutions (**first row**) and 0.5% *w*/*w* Mx-loaded 15% *w*/*w* Nc solutions (**second row**) in different solvents.

Nc dissolves easily in NMP and DMSO, while it dissolves less readily in Gf compared to Py. Figure 2 displays the physical appearance of 15% *w*/*w* Nc solutions and 0.5% *w*/*w* Mx-loaded 15% *w*/*w* Nc solutions in various solvents. All solutions appeared clear. Prepared solutions in Py, DMSO, and Gf exhibited a yellowish tint. The flowability of the solutions varied depending on the solvents used and correlated directly with the viscosity results.

### 2.1. Viscosity, Rheological Behavior, and Injectability

**Figure 3:**
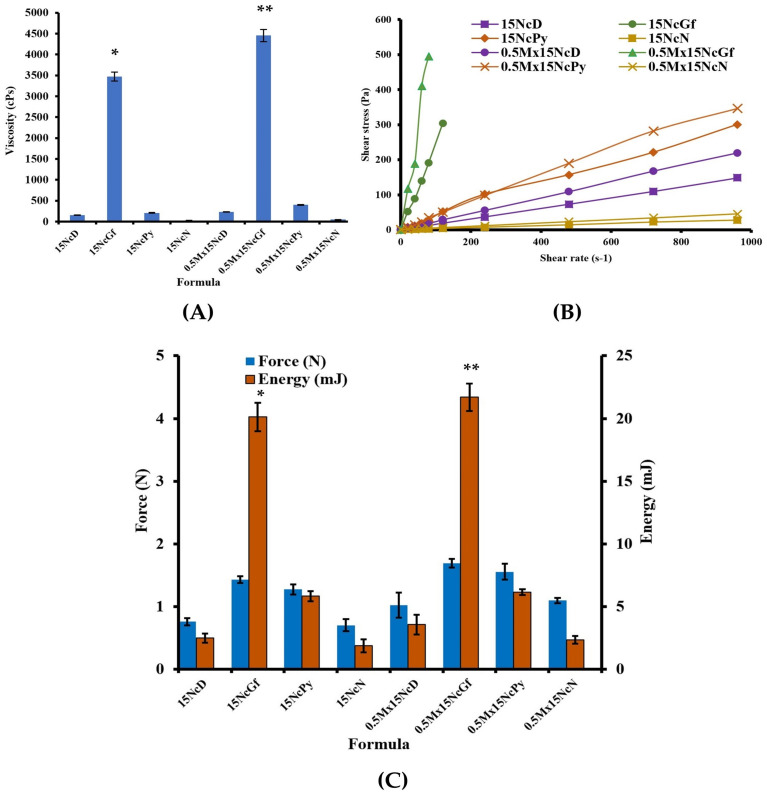
Viscosity (**A**); relationship between shear stress and shear rates (**B**); injection force and energy from injectability test (**C**) of drug free and Mx-incorporated Nc-based ISGs. * and ** represent a significant difference (*p* < 0.01) within the tested formulations.

Figure 3A illustrates the impact of different solvents on the viscosity of Nc solutions and Mx-loaded Nc solutions. When NMP was used as the solvent, the viscosity of both solutions was lower compared to DMSO, Py, and Gf. Notably, when Gf was used as the solvent for drug-free and drug-loaded Nc solutions, a significantly higher viscosity (*p* < 0.01) was observed. The viscosities of DMSO, Gf, Py, and NMP were recorded as 4.52 ± 0.01, 17.20 ± 0.20, 13.76 ± 0.20, and 4.88 ± 0.20 cPs, respectively. Hence, the choice of solvent had an influence on the resulting viscosity of the prepared ISGs. Typically, a solvent with good affinity for dissolving the compound can reduce the solution’s viscosity because the substance–solvent interaction dominates over substance–substance interaction [41]. The addition of Mx evidently increased the viscosity of all ISG solutions due to the replacement of solvent by dissolved drug molecules.

All of them showed the Newtonian flow behavior with linear relationship between shear stress and shear rate, as presented in Figure 3B. The greater the 3D network formation within the Nc molecule, the more it provoked a remarkable enhancement in the viscosity of these formulations. Due to their Newtonian flow behaviors, physicians and dentists have deemed them suitable for injection dosage forms, as the formulations can be injected through a needle by applying force to a syringe plunger, expelling the formulation through the stainless needle [42]. Comparing the results at a polymer concentration of 15% *w*/*w*, the shear stress and the viscosity of the Nc solution in NMP were found to be similar to those of ethyl cellulose and higher than those of Eudragit RS and bleached shellac ISGs in NMP, as previously reported [13].

The injectability of the fluid through a syringe and needle, as measured by the force and energy required, is presented in Figure 3C. The choice of solvent had a significant impact on the maximum force and energy needed for injection. All formulations were deemed acceptable for use as injectable dosage forms since the applied force remained below 50 N [43]. Specifically, the maximum force and the energy required for the injection of drug-free and drug-incorporated Nc solutions in Gf were significantly higher (*p* < 0.01) compared to those dissolved in Py, DMSO, and NMP. This finding aligns with previous results showing higher values for zein ISG in Gf compared to DMSO [44]. The energy required for the injection of these Nc ISG solutions was lower than that for polymeric ISGs containing 15% *w*/*w* bleached shellac (11.73 mJ) and 15% *w*/*w* Eudragit RS (8.04 mJ) [13]. The maximum injection force for all ISGs investigated in this study was below 5 N, indicating ease of injection and acceptability in terms of reduced discomfort at the injection site [42]. Therefore, all prepared drug-free and Mx-incorporated Nc-based ISGs were considered suitable for use as injectable drug delivery systems, offering convenient administration and satisfactory patient comfort and compliance [42].

### 2.2. Water Tolerance

**Figure 4:**
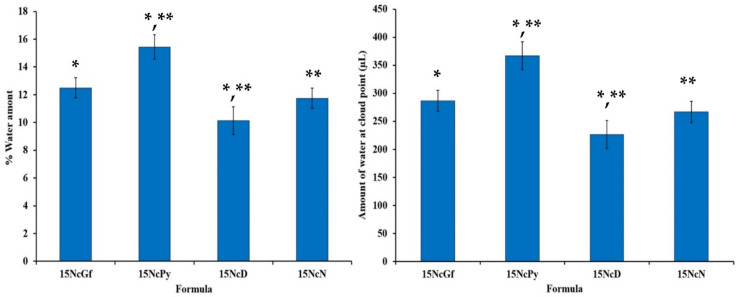
% Water amount and amount of water at cloud point of drug-free and Mx-incorporated Nc-based solutions after titration with deionized water at temperature of 25 °C (*n* = 3). * and ** represent a significant difference (*p* < 0.05) within the tested formulations.

The initial phase separation of a hydrophobic polymer, such as Nc, from the ISGs through solvent exchange with water can be detected by the turbidity observed when titrating ISGs with distilled water [32,33]. The water tolerance of ISGs, indicating the ability to withstand water without phase separation, can be assessed by the amount of water at the cloud point or the low percentage of water used. A lower water tolerance signifies a smaller aqueous phase inducing phase separation or a lower capacity of the ISG to tolerate water. The water tolerances of drug-free ISGs are presented in Figure 4. The Nc ISGs in DMSO exhibited significantly lower water tolerance (*p* < 0.05) compared to NMP and Py. The % water amounts inducing phase separation of 0.5Mx15NcGf, 0.5Mx15NcPy, 0.5Mx15NcD, and 0.5Mx15NcN were 12.50 ± 0.72, 15.44 ± 0.89, 10.14 ± 1.06, and 11.73 ± 0.74%, respectively. However, this did not show a significant difference compared to NMP, although it exhibited a tendency to be lower. In DMSO systems, fatty-acid-based ISGs, compared to systems using NMP as a solvent, required less water to reach the endpoint due to the higher water miscibility of DMSO, resulting from its higher polarity [45]. This higher polarity of water compared to the solvent in the ISGs led to the rapid phase separation of the fatty acid from DMSO as the aqueous phase gradually entered the ISG system, enhancing its polarity. As a result, the dissolved Nc could no longer withstand the system and separated into a cloudy mass. The decrease in water tolerance due to higher loading of matrix-forming agents has also been reported for ibuprofen-based ISGs [46] and fatty-acid-based ISGs [32,33]. Comparatively, all Nc ISGs exhibited higher sensitivity to water compared to ibuprofen-based ISGs because the Nc macromolecule was more prone to separation from the solvent induced by a non-solvent, unlike small molecules. The small quantity of this injectable delivery system could come into contact with and be exposed to crevicular fluid in the periodontal pocket, which could trigger phase separation. It is important to note that the water tolerances of the ISGs were below 18% *w*/*w* (Figure 4), indicating that the system could easily reach its saturation point due to the increasing hydrophilicity of the system and the low aqueous solubility of Nc.

### 2.3. Gel Formation

**Figure 5:**
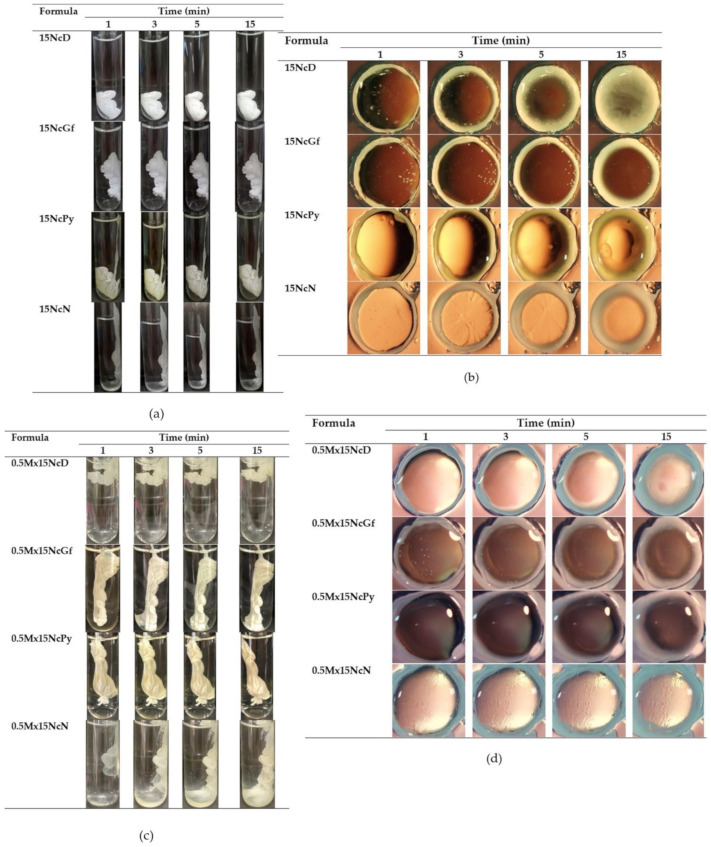
Gel formation after injection of the formulations into the phosphate buffer pH 6.8 (**a**) and after contact with agarose gel (**b**) of drug-free Nc-based ISG formulations, and gel formation after injection of the formulations into the phosphate buffer pH 6.8 (**c**) and after contact with agarose gel (**d**) of Mx-loaded Nc-based ISGs with different time intervals under stereomicroscope.

The prepared Nc-based ISGs underwent a transformation from a solution to an opaque mass and formed a ring when injected into PBS in a test tube and PBS-containing agarose wells, as shown in Figure 5. The presence of the opaque mass indicated the phase inversion of Nc upon exposure to water in PBS and agarose. Similar observations of opaque solid-like formations have been reported for natural-resin-based ISGs [12,14] and bleached-shellac-based ISGs [13,16] when exposed to an aqueous phase, owing to their rapid phase transformation [46]. In this study, the choice of solvent influenced the gel formation behavior. The Nc in DMSO formulations exhibited a noticeably rapid transformation. DMSO has higher polarity and water miscibility compared to other solvents, resulting in faster water diffusion and inducing solvent exchange [45]. Furthermore, as mentioned earlier, the high aqueous sensitivity of Nc accelerated this phase separation. The relatively quick transformation of all Nc-ISGs during injection makes it more practical for periodontitis treatment, improves patient compliance, and prevents formula leaks from the periodontal pocket [47]. However, it is crucial to ensure their ability for controlled drug release and antimicrobial activities, especially against periodontitis pathogens.

### 2.4. In Vitro Drug Release Study

**Figure 6:**
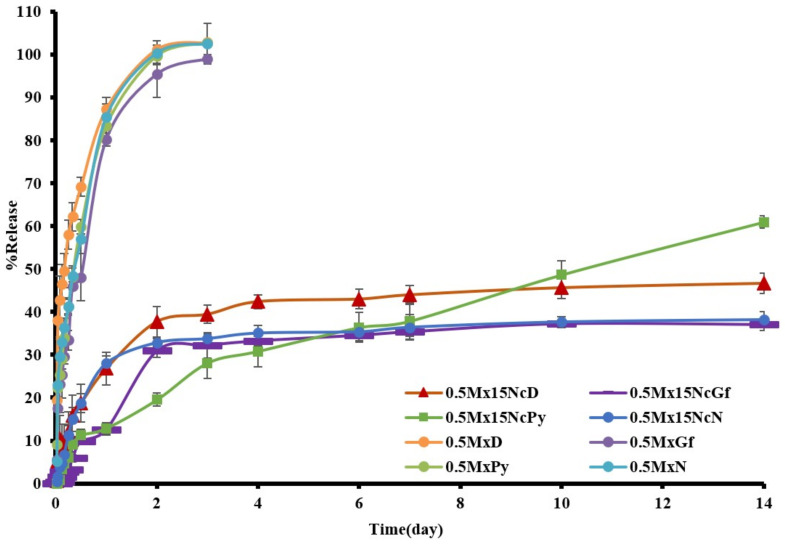
Release of Mx from Nc-based ISGs formulations using cup method (*n* = 3).

The drug content in samples of Mx dissolved in different solvents, including 0.5MxN, 0.5MxPy, 0.5MxGf, and 0.5MxD, was found to be 98.27 ± 1.01%, 97.61 ± 0.33%, 99.05 ± 1.06%, and 100.71 ± 0.83%, respectively. Meanwhile, the drug content in drug-incorporated ISGs, including 0.5Mx15NcN, 0.5Mx15NcPy, 0.5Mx15NcGf, and 0.5Mx15NcD, was 96.46 ± 0.86%, 98.11 ± 0.82%, 97.43 ± 1.27%, and 96.45 ± 0.52%, respectively. The release of Mx from the solutions without Nc addition was rapid, reaching a plateau state within 3 h (Figure 6). In contrast, a slower drug release was observed for all Mx-loaded Nc-based ISGs, and the burst drug release was reduced by incorporating Nc into the ISGs. The drug-incorporated Nc-based ISGs exhibited gradual release of Mx in the initial phase of the release profiles. Notably, 0.5Mx15NcPy showed a steeper release at a later stage, indicating a more pronounced and freely releasing behavior compared to other ISGs. This behavior can be attributed to the increased pore formation observed in the SEM analysis for 0.5Mx15NcPy. The 15% bleached-shellac-based ISG demonstrated sustained drug release of approximately 70% over 54 h, where the polymer concentration plays a crucial role in controlling the drug release mechanism [13]. The addition of a water-soluble additive has been investigated to modulate the drug release profile. For example, the addition of PEG 1500 reduced burst release and enhanced sustained release in a Eudragit^®^ RS ISG [48]. The inclusion of freely water-soluble compounds can facilitate drug release by creating high porosity, leading to increased erosion and subsequent drug release. Commercial products such as Atridox^®^ containing doxycycline hyclate using poly(D,L-lactide) as a polymeric material controlled the drug release for 1–2 weeks [17].

The fitting of drug release profiles, as shown in Table 1, indicated that all ISGs exhibited diffusion-controlled drug release, as they fit well with Higuchi’s equation and the n value estimated from the Korsmeyer–Peppas equation closely resembled the Fickian diffusion mechanism. Therefore, Mx diffused through the Nc mass after phase inversion into a gel state, and it gradually transformed into a more solid-like matrix over time as the solvent content in the ISG decreased. The steeper drug release observed in the initial phase may be attributed to drug release through the gel state before a slower release through the more solid-like matrix in the later phase of the release profile. A similar release pattern has been reported for doxycycline hyclate and vancomycin HCl released from natural-resin-based ISGs [14,49]. The process of drug release should be irreversible delivery because the transformed Nc mass could not dissolve in the release medium.

### 2.5. Scanning Electron Microscopy (SEM)

**Figure 7:**
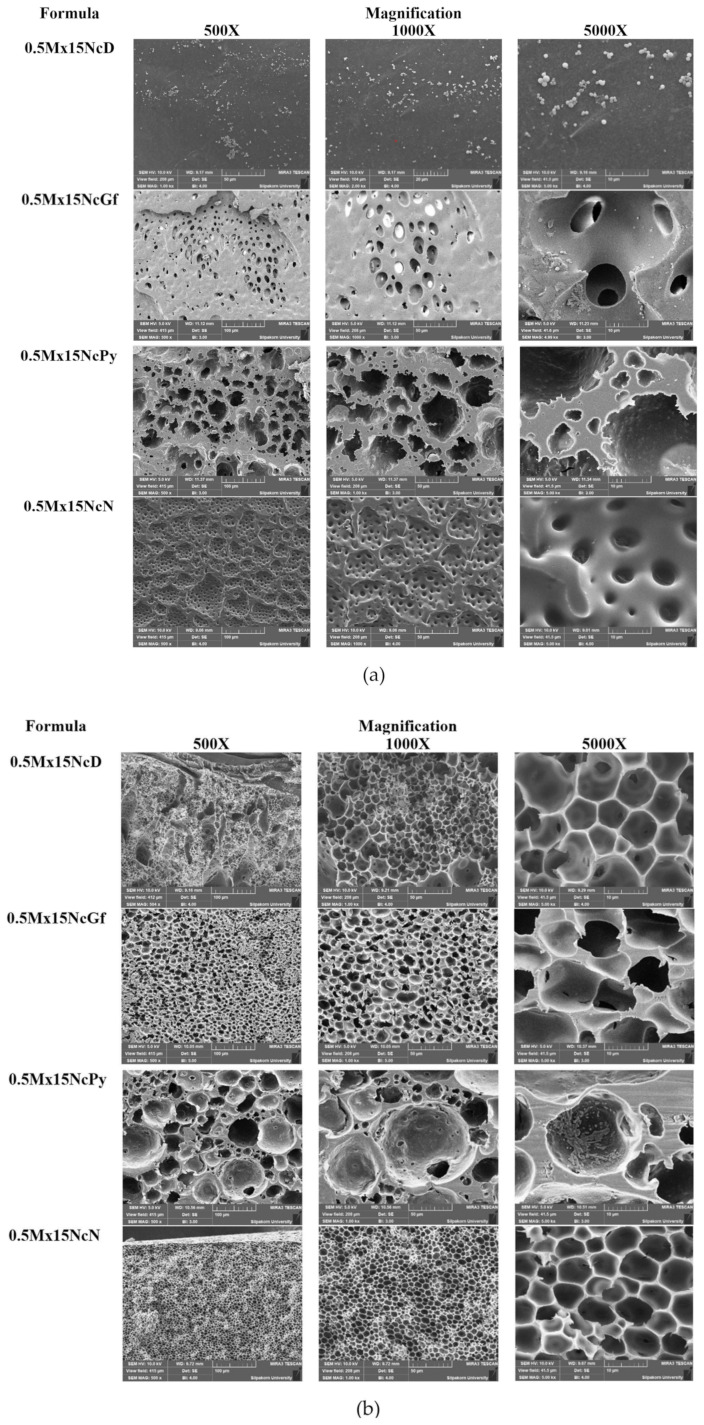
SEM images of surface (**a**) and cross section (**b**) of freeze-dried Mx-loaded Nc-based ISGs at different magnifications (500, 1000, and 5000×).

Figure 7 presents the SEM photographs depicting the surface topography of Mx-loaded Nc-based ISGs prepared using different solvents. The SEM images clearly show that the morphology of these ISGs varied significantly depending on the solvent used. The type of solvent had a marked influence on the surface topography, as shown in Figure 7a. The remnant of 0.5Mx15NcD, after 2 weeks of drug release, exhibited a relatively smooth surface without any visible pores. On the other hand, the remnants of 0.5Mx15NcN and 0.5Mx15NcGf showed a distinct rough and wavy surface with various open pores. The surface of 0.5Mx15NcN had a more homogeneous distribution of open pores compared to 0.5Mx15NcGf. Notably, larger open pores were observed on the remnant surface of 0.5Mx15NcPy. Cross-sectional views revealed an interconnected porous structure in these ISGs, as shown in Figure 7b. The presence of a more porous structure on the surface remnants was related to the increased drug release observed in 0.5Mx15NcGf, as mentioned earlier. No remaining Mx crystals were observed in the remnants, indicating that the drug release was nearly complete. When the ISG solutions came into contact with an aqueous solution, solvent diffusion into the aqueous phase occurred at different rates through solvent exchange. This subsequent phase separation of Nc led to the immediate formation of a sponge-like structure with a porous surface [44].

In comparison, the topography of the Nc sponge exhibited a more homogeneous and interconnected cell structure compared to the zein sponge-like ISG remnant reported in previous studies [44]. The presence of pores in the sponge structure served as channels for the release of drug molecules from the inner gel. The formation of pores in ISGs is typically influenced by the rate of solvent exchange [44,49]. Studies on propolis and benzoin-based ISGs have shown that slower gel formation rates result in higher drug release, while benzoin-based ISGs with hydrophobic characteristics are more suitable for modulating drug release [49]. Therefore, the characteristics of gel formation and the rate of solvent exchange directly affect both drug release and the sponge-like topography of ISGs.

When ISGs come into contact with an aqueous phase or body fluids, a phase inversion process is triggered in the Nc solutions, leading to the separation of the polymer into a gel state and eventually forming a solid matrix-like mass. This process is similar to the critical physicochemical parameters involved in modulating drug release from PLGA in situ implants, as described by Parent et al. [50]. The same components used in ISGs can be applied to the fabrication of in situ forming scaffolds, which are commonly used in tissue engineering applications [50,51]. The microphotographs of the remnants after the release test reveal the presence of pores formed within the matrix, indicating that solvent exchange plays a crucial role in modulating drug release from Nc-based ISG matrices.

### 2.6. X-ray Tomographic Imaging

**Figure 8:**
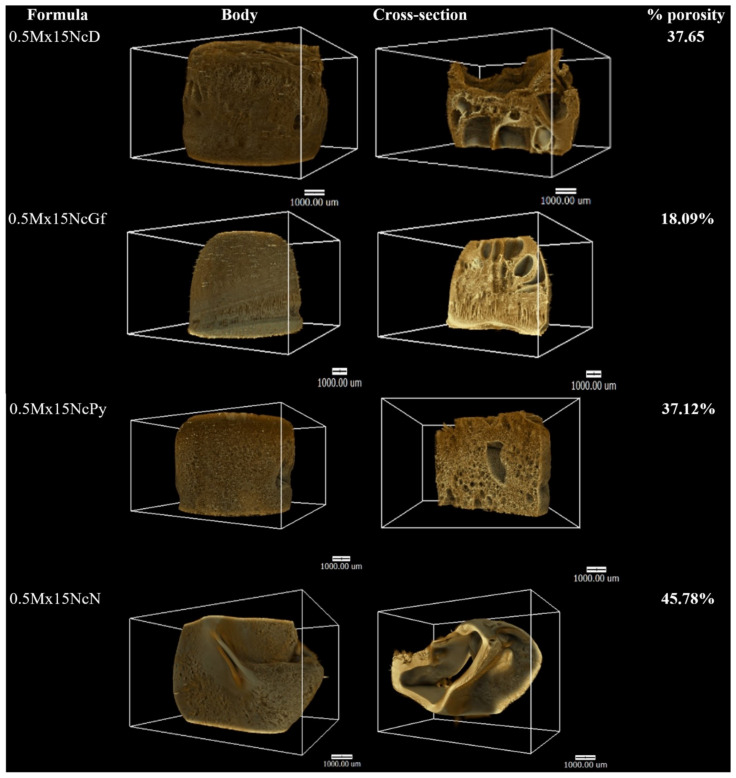
X-ray tomography image and %porosity using X-ray tomography of freeze-dried Mx-incorporated Nc-based ISGs.

X-ray computed tomography (CT) is a non-destructive imaging technique that can provide three-dimensional information about the internal details and properties of objects [52]. In this study, X-ray tomographic images of the remnants of Mx-loaded Nc-based ISGs obtained from a synchrotron light source are shown in Figure 8. The images depict the 3D volume of the remnants on the left side, while the cross sections on the right side reveal the presence of pores inside the remnants and provide information about their porosity. The use of X-ray tomographic imaging in this study offered several advantages over SEM. While SEM provides detailed information about specific areas, X-ray tomography allows for a comprehensive observation of the entire remnant structure. The images obtained from X-ray tomography confirmed the sponge-like topography of the remnants, but also provided insights into their internal structures, which may not be fully captured by SEM.

The remnant porosity of 0.5Mx15NcN was found to be greater than that of 0.5Mx15NcD, 0.5Mx15NcPy, and 0.5Mx15NcGf, as shown in Figure 8. This difference in porosity could be attributed to structural changes that occurred during the freeze-drying process. The presence of macrovoids or finger-like structures within the remnants was observed, resembling the structure of an asymmetric membrane with a thin top skin layer supported by a finger-like sublayer. The formation of finger-like structures typically involves two processes: pore initiation and growth [53]. During the liquid–liquid phase separation of the Nc-based solution, the development of Nc-rich and Nc-lean phases leads to the initiation of nuclei for the finger-like structures. These nuclei are mainly formed beneath the top layer, and their growth depends on the state of the solution at the interface of the phase separation [54]. The self-formation of scaffold structures is a characteristic feature of injectable in situ forming bioactive-compound-delivery systems. Therefore, X-ray tomographic imaging serves as a valuable technique for scrutinizing and tracking the three-dimensional geometries and porosity characteristics of these systems. It provides important insights into the internal structure and porosity of the remnants, aiding in the understanding and characterization of the Nc-based ISGs.

### 2.7. In Vitro Degradability

**Figure 9:**
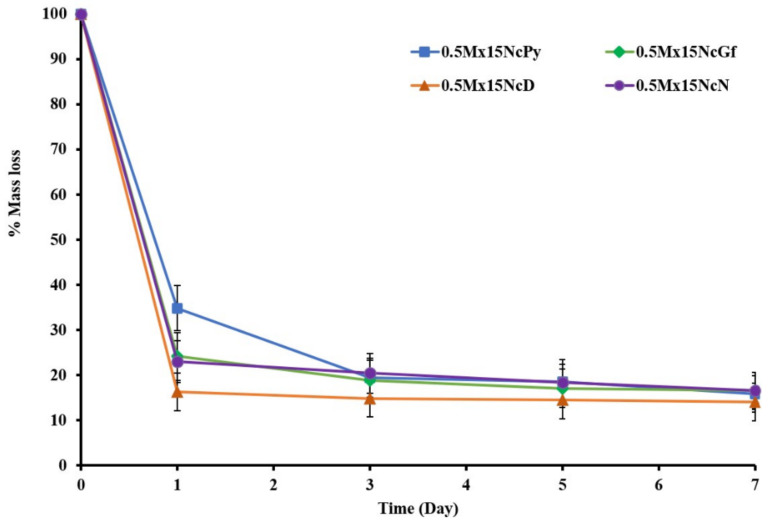
% Mass loss of Mx-loaded Nc-based ISGs (*n* = 3).

This study aimed to investigate the in vitro degradation behavior of the Mx-incorporated Nc-based ISGs by determining the mass loss. The percentage of in vitro degradation for the ISGs over one week is shown in Figure 9, and the values for %w_ex_ and %w_obt_ are calculated and presented in Table 2. The mass loss of the ISGs was assessed after incubation in a release medium at a specific pH, which is a common method for evaluating in vitro degradation [55]. An abrupt mass loss was observed for all ISGs on the first day, indicating the diffusion of both Mx and the solvent into the release medium through solvent exchange. As the release of the solvent and drug reached a steady state, the total mass loss of the ISGs gradually increased and eventually reached a plateau. Water uptake played a critical role in promoting this behavior. Among the ISGs, 0.5Mx15NcPy showed slightly lower % degradation compared to the other ISGs, which correlated with its lower drug release during the initial phase.

The degradation parameters %w_ex_ and %w_obt_ have been previously used to assess the in vitro degradation of Eudragit^®^ polymers-based ISGs for periodontal controlled drug delivery [56]. %w_ex_ represents the degradation under the assumption that the formed matrix was not degraded and the solvent was completely removed, including the weight of the released drug. On the other hand, %w_obt_ represents the degradation of the formed matrix and is expected to be lower than %w_ex_. A higher %w_obt_ compared to %w_ex_ indicates incomplete solvent exchange, suggesting that the system took a longer time to complete the solvent exchange process. In this study, the %w_obt_ values of all ISGs at the final time point were lower than %w_ex_, indicating that the formed matrix was degraded in all ISGs. The erosion of the Nc matrix from the scaffold may occur over time in the release medium, which further supports the degradation of the formed matrix. This result confirms the potential of this system for periodontal pocket drug delivery.

The degradation rate and degradation amount typically depend on the polymer content. The relatively low polymer content in these ISGs led to a higher degradation rate and greater degradation amount. For example, a commercial product like Atridox^®^ contains 37.5% PLA in NMP to control drug release [17]. The % degradation observed in this study was influenced by the loss of drug, solid matrix, and solvent content, which are related to the solubility of the drug, matrix, and miscibility of the solvent. Nc has been investigated for various applications, such as membrane immobilization of nucleic acids and proteins in blotting techniques, as well as wound-healing bandages, due to its non-toxic nature [7,9,10,11]. Therefore, Nc exhibits potential as an interesting polymer for use as the main component of ISGs for periodontitis treatment.

### 2.8. Antimicrobial Activities

**Figure 10:**
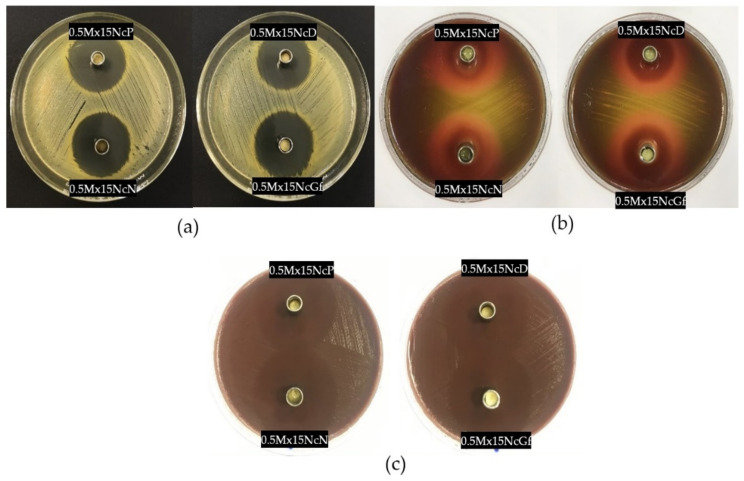
Photographs of inhibition zone of 0.5Mx15NcP (upper-left cup), 0.5Mx15NcD (upper-right cup), 0.5Mx15NcN (lower-left cup), and 0.5Mx15NcGf (lower-right cup) ISG formulations against *S. aureus* 43300 (MRSA) (**a**), *P. gingivalis* (**b**), and *A. actinomycetemcomitans* (**c**) (*n* = 3).

Table 3 shows the diameter of the zone of inhibition (ZOI) of solvents, Nc solutions, and drug-free and Mx-loaded Nc ISGs against *Staphylococcus aureus* ATCC 6538, *Staphylococcus* ATCC 43300 (MRSA), *Staphylococcus aureus* ATCC 6532, *Staphylococcus aureus* ATCC 25923, *A. actinomycetemcomitans* ATCC 29522, and *P. gingivalis* ATCC 33277. Examples of photographs from antimicrobial tests against *Staphylococcus* ATCC 4430 (MRSA), *P. gingivalis* ATCC 33277, and *A. actinomycetemcomitans* ATCC 29522 are shown in Figure 10. These microbe species are associated with periodontitis disease, especially pathogens such as *Porphyromonas gingivalis* and *A. actinomycetemcomitans*. *S. aureus* could be isolated from periodontitis [57] and it has the ability to form a biofilm which can lead to antibacterial drug resistance [58,59]. The organic solvents NMP, DMSO, Py, and Gf are used in the fabrication of depot dosage forms due to their safety and low toxicity [29,31,35,39]. In terms of their antimicrobial activity, Py exhibited higher activity against the tested microbes compared to NMP. Gf and DMSO also showed lower activities compared to Py and NMP, as indicated in Table 3. These solvents have the ability to dissolve the lipid components of bacterial cell envelopes, thereby disrupting the process of the nutrient transport of the bacteria. Consequently, these solvents are suitable for use in ISGs as delivery systems for antimicrobial compounds in the treatment of infectious diseases, as they can enhance the efficacy of antimicrobial activities. Additionally, their high permeability through cell membranes and their role as penetration enhancers [27,60,61] can further enhance the antimicrobial activities of antibacterial drugs.

The addition of Nc polymer to the solvents resulted in a decrease in the ZOI for all solvents, as shown in Table 3. This decrease can be attributed to the increased viscosity caused by the presence of Nc, as well as to the lack of inherent antibacterial activity of Nc. Many of the drug-free Nc-based ISGs exhibited significantly narrower ZOIs compared to their corresponding solvents (*p* < 0.05). The introduction of the dissolved Nc network in the ISGs effectively retarded the diffusion of solvents, leading to an increase in environmental viscosity and a consequent reduction in the ZOI.

However, the incorporation of the antibacterial drug Mx noticeably enhanced the antibacterial activities of both the polymer-free ISGs and the Nc-based ISGs. The addition of Nc significantly diminished the ZOI values of almost all prepared ISGs (*p* < 0.05). Despite this decrease, there was still a significant and relatively large ZOI observed against all tested microbes. This demonstrates that the use of Mx, a fourth-generation fluoroquinolone antibiotic, effectively inhibited the growth of the tested bacteria. Although bacteria are associated with periodontal disease, protection against inflammation and oxidative stress are related to the successful curative effect. Therefore, the loading of antimicrobials, antioxidants, and anti-inflammatory drugs with osteogenic active ingredients into drug delivery systems is currently interesting [62,63]. In addition, optimization in developing ISGs by employing design of experiments (DOE) should be further investigated [64]. Nowadays, many technologies such as foam engineering [65], metal-doped graphene models [66], metal-coupled fullerene synthesis [67], and nanocages should be applied in drug delivery systems such as ISGs for further investigations into their use as smart and targeted drug delivery processes [68]. Interest in engineered metal–organic frameworks (MOFs) with controlled sizes for biomedical applications has grown significantly in recent decades [69,70,71]. The tunable porosity, chemical composition, size, shape, and surface functionalization capabilities of MOFs make them increasingly attractive for drug delivery purposes; therefore, the application of MOFs as drug delivery features for ISGs should be of interest for further investigations.

## 3. Conclusions

Various solvents, including NMP, DMSO, Py, and Gf, were investigated regarding their effects on the physicochemical properties and bioactivity of Mx-incorporated aqueous-induced Nc-based ISGs for periodontal pocket delivery. The viscosity and the force required for injection of drug-free and Nc-incorporated ISGs prepared using Gf were notably higher than those prepared using Py, DMSO, and NMP as solvents. All of them exhibited Newtonian flow and transformed from a solution into a gel state, eventually forming a more solid-like matrix mass over time after exposure to the aqueous phase. The low injection force for all the ISGs indicated their ease of injection and acceptability, offering convenient administration and patient compliance. The Nc ISGs formulated in DMSO showed lower water tolerance or higher water sensitivity compared to NMP, Gf, and Py. Moreover, the Nc ISG in DMSO exhibited a noticeably rapid gel transformation. All the prepared Nc ISGs were actually water sensitive and underwent phase inversion from clear solution to form an opaque gel. Therefore, they demonstrated their ability to serve as aqueous-triggered in situ forming dosage forms. The obtained Nc mass effectively prolonged the liberation of Mx for two weeks from ISGs in simulated crevicular fluid through Fickian diffusion, while the initial burst release of the drug was reduced by incorporating Nc into the ISGs. SEM and X-ray imaging revealed that the different types of solvent promoted different sponge-like 3D structures and porosity in the dried NC scaffold due to solvent exchange with the release medium, along with simultaneous solvent diffusion outward, which accounted for the main mass loss during drug release. The incorporation of Nc reduced both solvent and drug diffusions, resulting in a narrower diameter of the zone of inhibition of bacterial growth. However, all the developed Mx-incorporated, Nc-based ISGs effectively inhibited the growth of four strains of *S. aureus*, including MRSA, and two pathogens associated with periodontitis, *A. actinomycetemcomitans* and *P. gingivalis*. As a result, the localized delivery of Mx through the fabricated Nc-based ISGs employing DMSO and NMP as the solvents, which transform into a gel upon aqueous induction and act as the Mx-incorporated Nc gels and subsequent matrices, allows for prolonged drug release with efficient antibacterial activities. Nevertheless, these Mx-incorporated aqueous-induced Nc-based ISGs need to be further investigated through clinical experiments on their efficacy and safety.

## 4. Materials and Methods

### 4.1. Materials

Nitrocellulose (Nc) (lot no. 200906/09018) (nitrogen content of 11.77% and acid content maximum of 0.03%) was graciously provided by Nitro Chemical Industry LTD, located in V.S. Chem House Bldg., Pathumwan, Bangkok, Thailand. Moxifloxacin HCl (Mx) (lot no. MN00000845) was generously supplied by Siam Pharmaceutical Co., also based in Bangkok, Thailand, and served as the model drug for the study. The solvents used included DMSO (≥99.9%, lot no. 1862992, Fisher Chemical, Horsham and Loughborough, UK), Gf (≥98.0%, lot no. BCCD7726, Sigma-Aldrich, Overijse, Belgium), PYR (≥98.0%, Fluka Analytical, Darmstadt, Germany), and NMP (≥99.5%, lot no. 144560-118, QReC, Auckland, New Zealand). Agarose (lot no. H7014714, Vivantis, Selangor Darul Ehsan, Malaysia) was utilized for analyzing gel formation behavior. The media used for antimicrobial investigations consisted of sheep blood agar and chocolate agar (Ministry of Public Health, Nonthaburi, Thailand), as well as tryptic soy agar and tryptic soy broth (DifcoTM, Detroit, MI, USA). Potassium dihydrogen orthophosphate (lot no. E23W60) and sodium hydroxide (lot no. AF310204) from Ajax Finechem, Seven Hills, NSW, Australia were employed as components in the preparation of phosphate-buffered saline (PBS). The test bacteria included *S. aureus* ATCC 6538, *S. aureus* ATCC 43300 (MRSA), *S. aureus* ATCC 6532, and *S. aureus* ATCC 25923 (Department of Medical Sciences, Ministry of Public Health, Nonthaburi, Thailand), as well as *A. actinomycetemcomitans* ATCC 29522 and *P. gingivalis* ATCC 33277 (MicroBiologics Inc., St Cloud, MN, USA).

### 4.2. Preparation of In Situ Forming Gel

ISGs were prepared by mixing various concentrations of Nc (15% *w*/*w*) dissolved in different solvents, namely, NMP (N), PYR (P), DMSO (D), and GF (G), using a magnetic stirrer for 120 min. Additionally, 0.5% *w*/*w* Mx was added to the Nc solutions. The formulation components are detailed in Table 4.

### 4.3. Rheology and Viscosity Characterization

Viscosity measurements were performed using a cone-plate viscometer model RM 100 CP2000 Plus (Lamy Rheology Instruments Company, Champagne-au-Mont-d’Or, France). The equilibration time for each measurement was set at 15 s. Rheological data was acquired using this specific instrument. The shear stress of the sample was determined across various shear rates, all maintained at room temperature. To ensure accuracy and reliability, all experiments were conducted in triplicate.

### 4.4. Injectability

To assess the injectability of the formulation, an injectability test was conducted. The compression mode of a texture analyzer (TA.XT plus, Stable Micro Systems, Godalming, UK) was employed for this purpose. The formulation was loaded into a 1 mL plastic syringe, which was connected to an 18-gauge stainless needle. The expelling force of the formulation from the syringe was determined using the texture analyzer. During the test, the upper probe of the instrument applied a constant force of 0.1 N and moved at a speed of 1.0 mm/s. The syringe plunger was gradually pressed by the probe until it reached the base of the syringe barrel. The maximum expelling force, indicative of the force required to inject the sample fluid through the needle, was recorded. Furthermore, the energy expended during the expulsion process was calculated based on the force-displacement profiles obtained. To ensure accuracy and reproducibility, the experiments were conducted in triplicate, thereby minimizing the potential for random variations and increasing the reliability of the results.

### 4.5. Water Tolerance Test

The evaluation of the ISG formulations’ ability to withstand water-induced phase separation was conducted to assess their capacity to maintain a stable solution state in the presence of water. For this purpose, a test was performed in which 20 μL of deionized water was incrementally added using a micropipette to 2.5 g of the sample contained in a glass test tube. The resulting mixture was then vigorously stirred using a vortex mixer until the initially clear ISG solutions became turbid due to the phase separation of borneol, indicating the onset of phase separation. The experiment was carried out at two different temperatures, 25 °C and 37 °C, representing typical environmental and physiological conditions, respectively. To quantify the water tolerance of the formulations, the amount of water that could be added before phase separation occurred was determined. This value, denoted as the water tolerance value, was calculated using the following equation (Equation (1)) (*n* = 3).

(1) $$\%\mathrm{water}\mathrm{tolerance}=\frac{\mathrm{water}\mathrm{amount}\left(g\right)}{\mathrm{sample}\mathrm{amount}\left(g\right)+\mathrm{water}\mathrm{amount}\left(g\right)}\times 100\%$$

### 4.6. Gel Formation Study

The gel formation was visually observed by injecting prepared formulations through a 1 mL syringe with an 18-gauge needle into a glass test tube containing PBS (pH 6.8). This investigation aimed to study the formation of the gel matrix over time, and photographs were taken at various intervals (0, 1, 5, 10, 20, and 30 min).

In addition, a 0.6% agarose solution was prepared by dissolving it in PBS (pH 6.8) and poured into petri dishes to create a gel with a height of 1 cm. Once the agarose gel had set, a cylindrical cup was used to create a 300 μL hole with a diameter of 7 mm at the center of the petri dish. This agarose gel with the hole represented an artificial crevicular pocket, simulating a human scenario. Next, a 150 μL portion of the ISG formulation was added into the cylindrical hole or well of the agarose gel. Upon contact with the aqueous phase of the agarose gel, the formulation underwent solvent exchange, resulting in a transformation into an opaque solid-like matrix through a phase inversion mechanism. The morphology of the matrix formation was documented using a stereo microscope (SZX10, Olympus Corp., Tokyo, Japan) at 1, 3, 5, 10, 15, 20, and 30 min. The evaluation of matrix formation was performed using SZX10 Series software under the stereoscope.

### 4.7. Drug Content and In Vitro Drug Release Studies

The drug content in the samples was quantified by employing a UV-spectrophotometer (Cary 60 UV-Vis, Model G6860A, Agilent, Petaling Jaya, Selangor, Malaysia) through the utilization of a standard curve. The UV-spectrophotometer operated at a wavelength of 288 nm, which provided optimal detection for the drug of interest. To ensure statistical reliability, the analysis was performed in six replicates. The drug release from the developed ISGs was tested and compared to a control formulation consisting of an antimicrobial drug dissolved in a solvent. To perform the drug release study, a cylindrical-shaped porcelain cup with a diameter of 1 cm and a height of 1.2 cm was filled with 0.4 g of the formulation. The cup was then immersed in 50 mL of PBS (pH 6.8) at a temperature of 37 °C. A rotation shaker was used to maintain a constant speed of 50 rpm, simulating the drug release behavior from a crevicular pocket. During the study, samples of the release medium (5 mL) were taken at specific time intervals, and an equal volume of fresh PBS was replenished. The concentration of the released drug was determined at a wavelength of 288 nm using a UV-Visible spectrophotometer (Cary 60 UV-Vis, Model G6860A, Agilent, Petaling Jaya, Selangor, Malaysia). The experiments were conducted in triplicate to ensure reliability and reproducibility of the results.

To determine the drug release mechanisms, mathematical models such as zero order, first order, Higuchi’s, and Korsmeyer–Peppas models were employed. The dissolution data obtained from the experiments were fitted to these models. The DD-Solver software application, which is an add-in program for Microsoft Excel (Redmond, King County, WA, USA) written in Visual Basic for Applications, was utilized to determine the release mechanism. The “n” value from the Korsmeyer–Peppas equation was used as an indicator of the drug release mechanism [54].

### 4.8. Scanning Electron Microscopy (SEM)

The morphology of the Nc ISG system was investigated using SEM (TESCAN MIRA3, Brno-Kohoutovice, Czech Republic) with an acceleration voltage of 15 kV. After conducting the drug release experiment in PBS with a pH of 6.8, the gel remnant was washed with 200 mL of distilled water and subsequently freeze-dried. The dried samples were kept in a desiccator for 72 h and then coated with gold prior to examination.

### 4.9. X-ray Imaging with X-ray Tomographic Microscopy

The remnants of the dried ISG, obtained after a 7-day drug release, were prepared using a freeze dryer (Triad^TM^ Labconco, Kansas City, MO, USA). They were then stored in a desiccator at room temperature for one week to prevent the melting and collapse of their structures. The prepared dried-ISG remnants were examined at the X-ray tomographic microscopy (XTM) beamline located at the Synchrotron Light Research Institute (SLRI) in Nakhon Ratchasima, Thailand. The synchrotron radiation X-rays were generated by a 2.2-T multipole wiggler in the Siam Photon Source, operating at 1.2 GV with a current of 150 mA. All the synchrotron radiation X-ray tomographic microscopy (SRXTM) experiments were conducted using a filtered polychromatic X-ray beam, with a mean energy of 12.5 kV, and a source-to-sample distance of 34 m. The X-ray projections of the samples were acquired using a detection system equipped with a 200 µm thick scintillator (YAG:Ce, Crytur, Turnov, Czech Republic), a lens-coupled X-ray microscope, and the sCMOs camera (pco. edge 5.5, 2560 × 2160 pixels, 16 bits) (Optique Peter, Lentilly, France). The tomographic scans were captured with an isotropic voxel size of 0.72 µm. After collecting the data, the X-ray projections were normalized using a flat-field correction algorithm and reconstructed using the Octopus reconstruction software (TESCAN, Gent, Belgium). Three-dimensional representations of the composite films’ tomographic volumes and segmentation analysis were performed using the Drishti software (National Computational Infrastructure’s VizLab, The Australian National University, ACT, Australia). The porosity of the samples was determined in three dimensions through segmentation analysis using Octopus Analysis (TESCAN, Gent, Belgium) [55].

### 4.10. In Vitro Degradability

In vitro degradation was investigated using mass loss determination with the direct contact method, as mentioned in the drug release test. The weight of sample at a specific time point was measured by subtracting the weight of cup, and the degradation parameter (*n* = 3) was calculated as follows:

(2) $$\%\;\mathrm{Degradation}=\left(\frac{W_{0}-W_{n}}{W_{0}}\right)\times 100$$

(3) $$\%\mathrm{Obtained}\mathrm{remaining}\mathrm{weight}\left(\%W_{obt}\right)=\frac{W_{n}}{W_{0}}\times 100$$

(4) $$\mathrm{Unreleased}\mathrm{Mx}=5-\left(\frac{5\times \%Drug\;release_{n}}{100}\right)$$

(5) $$\%\mathrm{Experimental}\mathrm{remaining}\mathrm{weight}\left(\%W_{ex}\right)=\%\mathrm{Nc}\mathrm{content}+\mathrm{Unreleased}\mathrm{Mx}.$$

where:

$W_{0}$ = weight of the weighted sample in the cup at beginning;
$W_{n}$ = weight of remaining sample at time n;
$\%Mx\;release_{n}$ = %Mx released at time n obtained from drug release study.

### 4.11. Antimicrobial Activities

The experiment utilized the following standard strains: *S. aureus* ATCC 6538, *S. aureus* ATCC 43300 (MRSA), *S. aureus* ATCC 6532, *S. aureus* ATCC 25923, *A. actinomycetemcomitans* ATCC 29522, and *P. gingivalis* ATCC 33277. To assess the antimicrobial properties of the ISG formulation, the researchers employed the agar diffusion assay, specifically, the cylinder plate method. This technique involved the diffusion of the formulation from a stainless-steel cylinder cup (with a diameter of 6 mm and a height of 10 mm) through agar gel that was inoculated with the respective microorganism being tested.

The bacterial and fungal cultures, with an approximate turbidity equivalent to the 0.5 McFarland standard, were spread on TSA. A 100 µL portion of each formulation was placed inside the cylinder cap, which was then positioned on the surface of the agar medium plate. In the case of *A. actinomycetemcomitans*, the bacterial culture with a turbidity matching the 0.5 McFarland standard was spread on chocolate agar. For *P. gingivalis*, the bacterial culture with a similar turbidity was spread on sheep blood agar. The entire experiment was conducted in an anaerobic incubator (Forma Anaerobic System, Thermo Scientific, Ohio, USA). Following incubation at 37 °C for 18 h, the diameter of the zone of inhibition (ZOI) against the respective tested organism was measured using a ruler. This process was repeated three times (*n* = 3). The test samples were solvents, Nc solutions, drug-free Nc-based ISGs, and Mx-incorporated Nc-based ISG formulations.

### 4.12. Statistical Analysis

The data obtained from the experiment were subjected to statistical analysis using the one-way analysis of variance (ANOVA) method, followed by the Tukey test for post-hoc comparisons. A significance level of *p* < 0.05 was used to determine statistical significance. The statistical analysis was performed using SPSS for Windows, specifically, version 11.5.

## Figures and Tables

**Table 1:** The regression coefficient (r^2^) values and diffusion exponent (n) values obtained from drug release profiles of Mx-incorporated Nc-based ISGs fitted to different mathematical equations.

Formula	Zero Order	First Order	Higuchi’s	Korsmeyer–Peppas		
	r^2^	r^2^	r^2^	r^2^	n	Release Mechanism
0.5Mx15NcD	0.6475	0.4541	0.8074	0.9155	0.298 ± 0.057	Fickian diffusion
0.5Mx15NcGf	0.7150	0.6826	0.8765	0.7805	0.278 ± 0.012	Fickian diffusion
0.5Mx15NcPy	0.9086	0.8892	0.9786	0.9705	0.458 ± 0.047	Fickian diffusion
0.5Mx15NcN	0.5725	0.3375	0.7547	0.8492	0.247 ± 0.053	Fickian diffusion

**Table 2:** Degradation parameters of Mx-incorporated Nc-based ISGs.

Day	% Obtained Remaining Weight (%w_obt_)				% Experimental Remaining Weight (%w_ex_)			
	0.5Mx15NcD	0.5Mx15NcGf	0.5Mx15NcPy	0.5Mx15NcN	0.5Mx15NcD	0.5Mx15NcGf	0.5Mx15NcPy	0.5Mx15NcN
1	14.2	21.7	26.9	17.5	21.6	27.2	33.7	28.7
3	13.6	18.2	17.6	16.8	15.2	23.4	29.2	22.4
5	13.8	16.3 *	17.0	15.4	13.4	20.5	25.1	20.7
7	12.5	14.6 *	16.2	15.2	13.1	19.6	22.6	20.9

* represents a significant difference (*p* < 0.05) within the tested formulations.

**Table 3:** Inhibition zone diameters of solvents, drug solutions, drug-free Nc-based ISGs, and Mx-incorporated Nc-based ISG formulations against various *S. aureus*, *P. gingivalis*, and *A. actinomycetemcomitans* (*n* = 3).

Formula	Inhibition Zone ± S.D. (mm)					
	*S. aureus* 6538	*S. aureus* 43,300 (MRSA)	*S. aureus* 6532	*S. aureus* 25,923	*P. gingivalis* ATCC 33,277	*A. actinomycetemcomitans* ATCC 29,522
NMP	15.0 ± 0.8	15.7 ± 0.5	14.0 ± 0.8	13.7 ± 0.5	15.7 ± 0.5	42.0 ± 1.6
2-PYR	17.7 ± 0.5	18.7 ± 0.9	16.7 ± 1.2	15.7 ± 0.5	20.7 ± 0.9	42.3 ± 0.5
DMSO	11.3 ± 0.5	12.0 ± 0.0	10.0 ± 0.8	9.7 ± 0.5	17.3 ± 0.5	26.3 ± 0.9
Glycerol formal	11.3 ± 0.5	11.3 ± 0.5	11.0 ± 0.8	13.3 ± 0.5	16.0 ± 0.8	32.0 ± 0.8
15NcN	11.3 ± 0.5 *	13.7 ± 1.2	14.0 ± 1.6	11.7 ± 1.2	12.0 ± 0.8	34.7 ± 0.9 *
15NcPy	12.0 ± 0.8 *	15.7 ± 1.2 *	13.7 ± 1.2	12.7 ± 0.5 *	13.7 ± 1.7 *	34.7 ± 1.2 *
15NcD	9.7 ± 0.5	10.0 ± 0.8	10.3 ± 0.5	- *	14.3 ± 1.2	21.3 ± 0.5 *
15NcGf	- *	12.0 ± 0.8	12.7 ± 1.2	10.0 ± 0.0	15.0 ± 0.8	29.7 ± 1.2 *
0.5MxN	32.0 ± 0.8	31.3 ± 0.5	33.0 ± 0.8	30.3 ± 0.5	29.0 ± 2.4	44.3 ± 0.5
0.5MxP	33.3 ± 0.5	32.3 ± 0.5	33.3 ± 0.9	32.7 ± 0.5	25.7 ± 0.9	45.0 ± 0.8
0.5MxG	37.7 ± 1.2	37.0 ± 0.8	38.0 ± 0.8	35.3 ± 0.5	34.7 ± 0.5	45.0 ± 0.8
0.5MxD	37.7 ± 0.5	37.7 ± 0.5	37.7 ± 0.9	35.3 ± 0.9	32.7 ± 0.9	45.7 ± 0.9
0.5Mx15NcN	28.7 ± 0.5 **	29.7 ± 1.2	30.0 ± 1.6	27.0 ± 0.8 **	25.7 ± 0.9	39.7 ± 0.5 **
0.5Mx15NcPy	26.7 ± 0.5 **	28.3 ± 0.5 **	28.0 ± 0.8 **	27.0 ± 0.0 **	27.0 ± 0.8	40.7 ± 0.5 **
0.5Mx15NcGf	32.7 ± 0.5 **	31.0 ± 0.0 **	32.7 ± 0.5	30.0 ± 0.0 **	26.7 ± 0.5 **	41.0 ± 0.8
0.5Mx15NcD	33.7 ± 0.5 **	31.7 ± 0.5 **	34.0 ± 0.8 **	29.3 ± 1.2 **	27.3 ± 0.9 **	43.3 ± 0.5 **

“-“ = no inhibition zone; the asterisk “*” indicates a significantly (*p* < 0.05) lesser inhibition zone diameter than that obtained using the pure solvent; the asterisks “**” indicate a significantly lesser inhibition zone diameter than that obtained using the drug-loaded solvent; established by using one-way ANOVA followed by an LSD post-hoc test.

**Table 4:** Composition of Mx-incorporated Nc-based ISG formulations.

Formula	Moxifloxacin HCl (Mx)	Nitrocellulose (N)	NMP	Py	DMSO	Gf
	(% *w*/*w*)	(% *w*/*w*)	(% *w*/*w*)	(% *w*/*w*)	(% *w*/*w*)	(% *w*/*w*)
15NcN	-	15	85	-	-	-
15NcPy	-	15	-	85	-	-
15NcD	-	15	-	-	85	-
15NcGf	-	15	-	-	-	85
0.5MxN	0.5	-	99.5	-	-	-
0.5MxPy	0.5	-	-	99.5	-	-
0.5MxGf	0.5	-	-	-	99.5	-
0.5MxD	0.5	-	-	-	-	99.5
0.5Mx15NcN	0.5	15	84.5	-	-	-
0.5Mx15NcPy	0.5	15	-	84.5	-	-
0.5Mx15NcGf	0.5	15	-	-	84.5	-
0.5Mx15NcD	0.5	15	-	-	-	84.5

## Data Availability

The data presented in this study are available on request from the corresponding author.
